# Hypertension, Diabetes Mellitus and Task Shifting in Their Management in Sub-Saharan Africa

**DOI:** 10.3390/ijerph7020353

**Published:** 2010-01-27

**Authors:** Alain Lekoubou, Paschal Awah, Leopold Fezeu, Eugene Sobngwi, Andre Pascal Kengne

**Affiliations:** 1 Department of Internal Medicine and Specialities, Faculty of Medicine and Biomedical Sciences, University of Yaounde I, Cameroon; E-Mails: lekoub77@yahoo.com (A.L.); sobngwieugene@yahoo.fr (E.S.); 2 Cerebrovascular Unit, Hôpital Neurologique Pierre Wertheimer, Hospices Civils de Lyon, France; 3 Department of Anthropology, Faculty of Arts, Letters and Social Sciences, University of Yaounde I, Cameroon; E-Mail: awahpaschal@yahoo.fr; 4 Department of Nutritional Epidemiology, French Institute of Health and Medical Research, Bobigny, France; E-Mail: leopoldfezeu@yahoo.fr; 5 The George Institute for International Health, University of Sydney, Australia

**Keywords:** chronic diseases, hypertension, diabetes, task shifting, nurse-led care, sub-Saharan Africa

## Abstract

Chronic diseases are becoming increasingly important in sub-Saharan Africa (SSA). The current density and distribution of health workforce suggest that SSA cannot respond to the growing demand for chronic disease care, together with the frequent infectious diseases. Innovative approaches are therefore needed to rapidly expand the health workforce. In this article, we discuss the evidences in support of nurse-led strategies for chronic disease management in SSA, with a focus on hypertension and diabetes mellitus.

## 1. Introduction

Sub-Saharan Africa (SSA) is currently going through demographic and health transitions characterized by more people living in urban areas and a mixed pattern of diseases occurrence with coexistence of both acute infections and chronic diseases. As a consequence, chronic conditions like hypertension and diabetes mellitus, often considered to be rare are now more prevalent in SSA and are fuelling a burgeoning burden of cardiovascular diseases, including ischemic heart diseases, strokes and peripheral arterial diseases [1,2]. A recent SSA study has clearly established the association between classical cardiovascular risk factors and all-cause mortality in rural Cameroon [3], therefore, stepping away from the old tradition of extrapolating from data sources beyond SSA. In this study age, male gender, smoking status, systolic blood pressure and fasting capillary glucose were independent predictors of 9-year all-cause mortality. This extension of the devastating consequences of chronic diseases to the rural underserved SSA populations indicates the need for urgent preventative solutions to advert the full development of an epidemic of chronic diseases. However, the current density and distribution of health workforce clearly indicate that SSA cannot cope with the growing need for care and prevention of chronic diseases together with the endemic infectious diseases. Innovative solutions are therefore required to quickly expand the health workforce in SSA and meet the demand to care for chronic disease. In this article, we discuss the growing evidence in support of nurse-led strategies for chronic disease management in SSA, with a focus on hypertension and diabetes mellitus.

## 2. The Burden of Hypertension and Diabetes in SSA

Shifts in hypertension proportionately reveal upward trends in both developed and developing countries. These trends shift further to the right comparatively for developing countries when they are plotted on graphs. Poulter and his collaborators have earlier observed a rightward shift of the blood pressure (BP) distribution curves among Kenyans who migrated from rural to urban areas [4]. The global burden of hypertension in 2000 and projection for 2025 have been recently evaluated by Kearney *et al.* [5] using data from national and regional surveys, indicating a higher absolute number of people living with hypertension in economically developing countries, including SSA. According to these authors [5], in 2000 the estimated total number of people with hypertension was 972 million (95% confidence interval: 957–987 million); 333 million (329–336 million) in economically developed countries, and 639 million (625–654 million) in economically developing countries. In the same report, the number of people with hypertension in economically developed countries was projected to increase by 24% from 333 million to 413 million (409–418 million), a rise of 80% was predicted for economically developing countries from 639 million to 1.15 billion (1.12–1.17 billion) [5]. The same disturbing data and trends apply to diabetes mellitus. The 4^th^ edition of the Diabetes Atlas of the International diabetes Federation provides recent figures of diabetes at the global, regional and country levels. Based on these estimates, there will be about 12.1 million individuals with diabetes in SSA in 2010. This figure is expected to increase by 98% so that by 2030, there will be about 23.9 million people with diabetes in SSA [6].

Fezeu *et al.* [7] described the temporal variation in BP and prevalence of hypertension in SSA based on contemporary diagnostic criteria. Between 1994 and 2003 in this study [7], there was a shift to the right of both cumulative curves of blood pressure, and the prevalence of hypertension increased by 2- to 5-fold in rural and urban Cameroonian men and women. More specifically, the age-standardized prevalence of hypertension changed from 20.1 % to 37.2% among women and from 24.4% to 39.6% among men. Over this ten-year period systolic (SBP) and diastolic (DBP) blood pressure levels significantly increased in rural women (SBP +18.2 mmHg, DBP +11.9 mmHg) and men (SBP +18.8 mmHg, DBP +11.6 mmHg), all p < 0.001. In the urban area, SBP increased in women (+8.1 mmHg, p < 0.001) and men (+6.5 mmHg, p < 0.001), and DBP increased only in women. Concerning diabetes mellitus in Cameroon, the age-standardized prevalence of diabetes in the rural and urban population ranged from 0.8 % to 1.6 % in 1997. More recently, examining 2,465 subjects aged 15 year and above, Sobngwi *et al.* reported a prevalence rate for diabetes mellitus across rural and urban areas ranging from 2.9% to 6.2% [8].

Mayosi *et al.*’s summary of health status in South Africa reported a rising burden of non-communicable diseases including diabetes and hypertension in rural communities as well as in urban areas, disproportionately affecting poor people living in urban settings. In that report, population-based surveys of the early 1990s showed a high prevalence of hypertension (14–33%) and diabetes (4.8–6%). In South Africa, sustained increases in premature adult deaths (15–64-year-olds) were seen for diabetes (38%) and hypertensive heart disease (20%) from 1999 to 2006 [9].

## 3. Task Shifting in Health Care

Task Shifting describes a situation where a task normally performed by a physician is transferred to a health professional with a different or lower level of education and training, or to a person specifically trained to perform a limited task only, without having a formal health education. One of the rationales for applying task shifting in developing countries is that, the alternative would be no care as heath systems are impeded by the growing health workforce shortage and imbalance in the distribution [10]. In its September 2004 report, the Human Development sector of the World Bank for the African region issued alarming figures on health workforce distribution in SSA [11]; the average ratio of physicians per 100,000 people in SSA was a meager 15.5, compared to an average of 311.0 in nine selected industrialized countries. For nurses, the same comparison was 73.4 in SSA and 737.5 in industrialized countries. These figures are unevenly distributed across the continent as shown in Table 1 [11]. One of the cornerstones of primary health care reforms advocated by the World Health Organisation in the 2008 world health report is achieving a universal health coverage and reducing health inequalities [12]. But the severe shortage and imbalanced distribution of trained health workforce poses a serious threat to achieving them at required scale and the set time. Although task shifting is an attractive solution to the shortage of health workforce, some caution is needed for its implementation. For instance, it should be applied in an organized manner so that the quality of care and patient safety is not compromised. Physicians should be consulted and the development of sustainable and fully functioning health care systems maintained. Task shifting should be preceded by a systematic review, analysis and discussion of the potential needs, costs and benefits. Research plays an important role in the success of this approach by helping to identifying successful training models, collecting and sharing information, evidence and outcomes [10,13]. In developing countries in general and in SSA in particular, task shifting has been used with compelling evidence on the feasibility and indicators of success for various disease entities and health sectors including human immunodeficiency virus/acquired immunodeficiency syndrome (HIV/AIDS), tuberculosis, mother and child health, and non communicable diseases [13–15]. Existing experience in the field of cardiovascular diseases, diabetes and hypertension may not be as extensive as that for communicable diseases but shades some light for a way forward. The following section is a summary review of such experience, with particular application to nurse-led care for diabetes and hypertension in SSA.

## 4. Nurse-Led Care for Hypertension and Diabetes in SSA

### 4.1. Data Source

A Medline search of the literature on task shifting and diabetes/hypertension in SSA was conducted by one co-author (AL) in October 2009. We initially used the terms “task shifting” and “Africa” which provided 29 entries. As this was judged to be insufficient because of the few number of articles, the search was subsequently extended using the combination of key words “doctor(s)-substitution” and “Africa” (six articles), “nurse(s)-substitution” and Africa (one article), “nurses-led care” and “Africa” (three articles), “community workers”, “diabetes” and “Africa” (two articles), “community workers”, “hypertension” and “Africa” (three articles), “nurse”, “diabetes”, “Africa” (26 articles), “nurse”, “hypertension” and “Africa” (31 articles), The titles and abstracts uncovered by these searches were reviewed and potentially relevant full text were retained for further evaluation. The full text references were checked for other potentially relevant articles. We retained articles written either in French or English. The studies eligible for the review included intervention studies with measurable longitudinal follow-up and outcomes. A total of five articles fulfilled the inclusion criteria. Of the five articles retained, there were three from South Africa and two from Cameroon. These articles are summarized in Table 2. No meta-analysis was conducted.

### 4.2. Nurses-Led Care for Hypertension

In a context of limited health workforce, several approaches have been used to improve access to care for hypertension around the world. A recent overview of relevant studies supports the promising role of nurse-led care for hypertension [21], which may therefore constitute an acceptable alternative in SSA where an acute shortage of trained physicians is being experienced. The nurses-led care approach to hypertension has been tested and assessed in some settings in SSA. In a retrospective review of medical registers of patients with stable hypertension followed-up by nurses in Harare municipal clinics, Basset *et al.* concluded that nurse-led care for hypertension was feasible [22].

More elaborate studies and particularly protocol driven nurse-led care with quantifiable measures of outcome have been reported in a few SSA Countries, including South Africa, where 68% of treated hypertensive patients had their BP controlled using nurse-led care in rural area [17]. In this pioneer prospective study of task-shifting in SSA, trained nurses were involved at the initial diagnosis step, management and follow-up of patients [17]. Kengne and his colleagues have implemented a nurse-led hypertension management protocol in rural and urban Cameroon [19]. In this prospective study that involved 454 patients, nurses received an initial and follow-up standardized training in five pilot clinics. Between baseline and final visits, SBP and DBP dropped by 11.7 (8.9−14.4) mmHg and 7.8 (5.9−9.6) mmHg, respectively [19]. This model of non-communicable diseases management by nurses has been duplicated in Ethiopia with good results although measurable outcomes were not provided by the authors [23].

### 4.3. Nurses-Led Care for Diabetes Mellitus in SSA

As it has been shown with hypertension, nurses-led care has been successfully implemented for diabetes mellitus in SSA. In an early experience in South Africa, nurses working alone achieved control of 82% of type 2 diabetic patients seeking care in their health facility, although this was based on a small number of participants (28 patients). In addition clinical criteria were used to assess the control of type 2 diabetes, essentially the presence/absence of symptoms of hyperglycemia or hypoglycemia, with no objective measure of blood glucose control [17]. More recently, a simple protocol and education-based diabetes care implemented by nurses in South Africa was associated with positive outcomes in term of glucose control [16]. In this study that involved 197 participants, the hemoglobin A_1_c (HbA1_C_) dropped from 11.6% (standard deviation = 4.5%) at baseline to 8.7% (2.3%) at 6 months and 7.7% (2.0%) after 18 months of follow-up [16]. In addition, subgroup analysis showed that education alone, regardless of the type of anti-diabetic agents or changes in their dosage, also improved glucose control (HbA1c dropped from 10.6% (4.2%) at baseline to 7.6% (2.3%) at 18 months). As part of the Essential non-communicable disease health intervention project (ENHIP), nurses-led diabetic clinics were set-up in rural and urban Cameroon [24]. Nurses were trained to deliver protocol driven glucose control to a total of 225 patients. After a follow-up duration of 1110 patient-months, there was a significant downward trend in fasting capillary glucose overall (p < 0.001) and in most subgroups of participants. Between baseline and final visits, mean fasting capillary glucose dropped by 1.6 (0.8−2.3) mmol/L. Among those with hypertension, blood pressure also decreased significantly [20].

## 5. Conclusions

The burden of chronic diseases in SSA is large, somewhat unique and growing, and the challenges to care and prevention are substantial. The shortage and misdistribution of health workforce in the region call for new strategies to quickly expand the workforce and cope with the high demand for care and prevention. This review suggests that task-shifting, which consist of relocating the tasks among available health care staffs has been implemented in few countries in the region with some indicators of success in the care of chronic diseases. Because nurses are likely available in most settings in Africa and in greater number compared with physicians, empowering the formers is a potential solution to the acute shortage of trained health staffs for the control and prevention of chronic diseases in SSA.

With regard to hypertension and diabetes mellitus, although still very limited, available experiences suggest that, acceptably designed interventions have clearly demonstrated the feasibility and utility of task-shifting for their care in SSA. The significant and measurable positive impact on surrogates of disease control confirms the potential of task-shifting as an alternative to rapidly increase the health workforce for hypertension and diabetes care in SSA. Interestingly, diabetes and hypertension management programs are usually complementary in the sense that the two conditions share similar risk factors and tend to cluster in the same individuals. In addition, in places in Africa where nurse-led care has been expanded to include other chronic diseases, similar positive outcomes have been reported for other chronic diseases [17,25,26]. For instance, the ENHIP program in Cameroon convincingly demonstrated that, with little additional training and supervision, and using simple clinical pathways, the same nurses were able to deliver a level of care that translated into improved outcomes for diabetes mellitus, hypertension, asthma and epilepsy in urban and rural settings [19,20,24–26]. Therefore, combining many chronic diseases in the same package delivered by nurses in SSA is achievable and will have added value in term of cost-effectiveness.

Experience from around the world suggests that task-shifting has been largely trialed and/or is the basis for providing care for diabetes and hypertension, particularly in primary care settings including general practice and community-based facilities [27–29]. In such settings, with regard to nurse-led care, there seems to be substantial variation in the task actually performed by primary care nurses, the level of responsibility assigned to them and the models they use in practice (‘partner in care’ *vs.* ‘assistant to the general practitioner’). With regard to the patient outcomes under nurse-led care elsewhere, there is evidence that nurses in primary care settings can provide effective care that translates into positive health outcomes for patients, similar to that provided by physicians [27]. Nurse-led care may achieve higher levels of patient satisfaction and better quality of life than physician-led care, which have been identified as areas of health care that require some improvement in Africa [27,30]. Globally, nurse-led care with particular application to chronic diseases is a relatively new and developing field, with nurses gradually assuming roles that have, hitherto, been the exclusivity of medical profession.

The present study has some limitations. Literature search and data extraction for the systematic review imbedded in the manuscript was conducted by one reviewer and limited to a single database. It is of note that all other co-authors were involved in previous studies of task-shifting in Cameroon, and involving them again in a systematic review relating to this topic in SSA could potentially bias the results. By searching only one database, it is possible that we have missed published studied that were not indexed to PubMed. Including such studies if any will likely strengthen the conclusions of the present study, unless the non included studies have systematically reported a harmful effect of nurse-led care for hypertension or diabetes, which is unlikely. There is no uniform definition of nurse-led care in the published literature, with substantial variations in the definitions across countries and studies. These variations could affect our chances of capturing all relevant studies, but again without affecting our conclusions. The present report is largely driven by studies conducted in Cameroon and South Africa which presently are the two countries in the region with documented experiences of task-shifting in the care of chronic diseases. It is expected that as more countries in the region will embrace this approach, more representative data will become available.

Existing experiences of task-shifting in chronic diseases care should be standardized and replicated in many settings in Africa alongside other suggested measures to improve access to prevention and care for these conditions [31]. In this line, recent developments suggest that nurse-led care for chronic diseases is being introduced to the training curriculum in a few SSA countries [32]. Collaborative approach between key players at various levels will have a greater impact. We also believe that these task shifting efforts should go beyond health workforce and focus on people with chronic diseases and their peers. To further develop this concept, we are currently piloting a research program in the North-West region of Cameroon where we are intending to use trained nurses and lay-people (family members and friends) as peer supporters to extend the education and follow up of patients with diabetes beyond clinical settings and improve adherence and treatment outcomes [33].

## Figures and Tables

**Table 1.** Distribution of health workforce (per 100,000 population) in sub-Saharan African countries [11].

**Countries**	**Physicians**	**Nurses**	**Midwives**	**Pharmacist**
Angola	5	114.0	4.3	NA
Benin	10.0	20.0	7.9	NA
Botswana	28.7	241.0	0.0	NA
Burkina Faso	4.0	26.0	3.4	NA
Burundi	0.5	1.0	NA	NA
Cameroon	7.4	36.7	0.5	
Cape Verde	17.1	55.8	NA	NA
Central African Republic	3.5	8.8	4.9	NA
Chad	2.5	15	2.3	NA
Congo	25.1	185.1	24.9	NA
Côte d’Ivoire	6.8	44.1	15.0	NA
Democratic republic of Congo	9.0	31.2	NA	NA
Djibouti	13.0	64.0	NA	2.0
Eritrea	5.1	21.0	2.2	
Ethiopia	3.0	6.0	NA	NA
Gambia	3.5	12.5	8.2	NA
Ghana	9.0	64.0	53.2	NA
Guinea	13.0	55.7	5.2	NA
Guinea Bissau	16.6	109.3	12.7	NA
Kenya	14.1	108	NA	NA
Lesotho	7.0	33.0	47.0	NA
Liberia	2.3	5.8	4.3	NA
Madagascar	8.7	18.8	10.7	NA
Mali	4.4	12.6	3.0	NA
Mauritania	13.8	62.4	10.1	NA
Mauritius	85	232.9	NA	NA
Mozambique	2.4	20.5	NA	NA
Namibia	29.1	165.8	116.5	NA
Niger	3.3	23.1	5.5	NA
Nigeria	26.9	66.2	52.4	NA
Sao Tome and Principe	46.7	127.4	29.6	NA
Senegal	10.0	50.0	6.6	NA
Seychelles	132.4	467.6	394.6	NA
Sierra Leone	8.8	90.7	4.7	NA
Somalia	4.0	20.0	NA	0.1
South Africa	25.1	140.0	NA	NA
Sudan	16.0	86.0	NA	1.1
Swaziland	15.1	40	NA	NA
Tanzania	4.1	85.2	44.8	NA
Togo	5.6	16.7	10.4	NA
Uganda	4.7	5.6	13.6	NA
Zambia	6.9	113.1	NA	NA
Zimbabwe	5.7	54.1	28.1	NA
**African region Average**	**25.1**	**93.5**	**30.9**	**NA**

**Table 2.** Summary of studies on task shifting in SSA applied to Diabetes mellitus and Hypertension.

**Country**	**First author**	**Year published**	**Setting**	**Study design**	**condition addressed**	**Number of participants**	**Task-shifting pattern**	**Main findings**
South Africa	Gill GV [16]	2008	Rural	Interventional Nurse-led protocol and education based	Diabetes	980 including 284 selected for analysis	To nurses	HbA1c was 11.6 ± 4.5% at baseline, 8.7 ± 2.3% at 6 months and 7.7 ± 2.0% at 18 months Education alone improved HbA 1c From 10.6 ± 4.2% baseline to 7.6 ± 2.3% at 18 months
South Africa	Coleman R [17]	1998	Rural and urban	Interventional Nurses-led protocol	diabetes and hypertension	713 including 165 selected for analysis (hypertension) 188 including 28 selected for analysis (Diabetes)	To nurses	68% of hypertensive “controlled” 82% of diabetic “(asymptomatic)
South Africa	Bradley HA [18]	2007	Urban	Interventional	Diabetes and hypertension	N/A (community based)	Community health workers	N/A
Cameroon	Kengne AP [19]	2009	Rural and urban	Interventional Nurses-led protocol	Hypertension	454	To nurses	The mean changes in SBP between first and last visit was −11.7 mmHg (P < 0.001) and in −7.8 mm in DBP (P < 0.001)
Cameroon	Kengne AP [20]	2009	Rural and urban	Interventional Nurses-led protocol	Diabetes	225	To nurses	Between baseline and final visits, mean fasting capillary glucose dropped by 1.6 mmol/L (95% CI: 0.8–2.3; *p* ≤ 0.001).)
